# The Effect of Tocilizumab on Inflammatory Markers in Patients Hospitalized with Serious Infections. Case Series and Review of Literature

**DOI:** 10.3390/life11030258

**Published:** 2021-03-20

**Authors:** Mark Berman, Ronen Ben-Ami, Shlomo Berliner, Marina Anouk, Ilana Kaufman, Adi Broyde, Sara Borok, Ori Elkayam

**Affiliations:** 1 Department of Rheumatology, Sackler Faculty of Medicine, Tel Aviv University, Tel Aviv 69978, Israel; marinaano@tlvmc.gov.il (M.A.); ilanak@tlvmc.gov.il (I.K.); adibro@tlvmc.gov.il (A.B.); sarabo@tlvmc.gov.il (S.B.); orie@tlvmc.gov.il (O.E.); 2 Infectious Disease Unit, Sackler Faculty of Medicine, Tel Aviv University, Tel Aviv 69978, Israel; ronenba@tlvmc.gov.il; 3 Tel Aviv Medical Center, Internal Medicine E Department, Sackler Faculty of Medicine, Tel Aviv University, Tel Aviv 69978, Israel; berliners@tlvmc.gov.il

**Keywords:** tocilizumab, infection, rheumatoid arthritis, giant cell arteritis, C-reactive protein, inflammation

## Abstract

Background: The human anti-IL-6 receptor antibody tocilizumab (TCZ) has been approved for the treatment of rheumatoid arthritis (RA) and giant cell arteritis (GCA). It is observed that CRP levels drop quickly after starting TCZ treatment. This may lead to misinterpretation of laboratory results when accessing the patient with infectious disease while on TCZ. We conducted this study to report cases treated with tocilizumab who developed serious infections with special reference to levels of CRP and to review the literature on the effect of tocilizumab on acute phase response (APR) during infections. Methods: The files of RA and GCA patients hospitalized in the Tel Aviv medical center between 2009–2019 were reviewed. Cases of patients with RA and GCA treated with tocilizumab who were hospitalized due to severe infections were reviewed with special emphasis on the duration of treatment, type of infection, and APR. Results: We identified nine admissions. Seven patients were treated with tocilizumab for RA, two for GCA. The diagnosis was pneumonia in three cases, osteomyelitis in one, cellulitis in one, endocarditis due to Whipple disease in one, abscess of cervix uteri in one, meningitis in one, and perforated diverticulitis in one. The mean CRP levels on admission were 4.75 mg/L (normal range, up to 5 mg/L). All cases were diagnosed correctly on admission. Conclusions: CRP levels may not correctly reflect the severity of infectious diseases during tocilizumab treatment. Increased awareness of the masking effect of tocilizumab on the APR during infection is needed in order to avoid a delay in the diagnosis.

## 1. Introduction

The human anti-IL-6 receptor (IL-6R) antibody tocilizumab (TCZ) has been approved for the treatment of rheumatoid arthritis (RA) in patients who respond inadequately or are intolerant to therapy with disease-modifying antirheumatic drugs (DMARDs). It has also been recently approved for the treatment of giant cell arteritis (GCA). It is a humanized monoclonal antibody targeting interleukin-6 (IL-6) receptors, blocking the pro-inflammatory effects of IL-6 and affecting the function of neutrophils, T cells, B cells, monocytes, and osteoclasts [1,2,3]. Treatment with TCZ is generally considered to be well tolerated, however, it may be associated with an elevation of liver enzymes and serum cholesterol levels, as well as an increased rate of infections [4].

IL-6 is a pro-inflammatory cytokine that is synthesized in the initial stages of inflammation. It induces the hepatic acute-phase response and mediates the production of C-reactive protein (CRP) during infection [5]. It also plays an important role in immune responses and is implicated in the pathogenesis of many autoimmune diseases. There is a clear correlation between increasing levels of IL-6 during inflammation and levels of CRP [6], with IL-6 being the main inducer of the CRP gene [7].

CRP, a 23-kDa protein named according to its ability to bind proteoglycan C of pneumococci, is part of an old defense system against microbes. CRP is predominantly produced by the liver and rises up to 100-fold upon challenge following inflammatory stimuli. It is thought that most of the interaction between CRP and the immune response to pathogens involves the binding of CRP to phosphocholine and the activation of the classical complement pathway [8]. Several studies have shown that CRP may provide protection against infection by the gram-positive pathogen Streptococcus pneumoniae [9], Salmonella enterica serovar Typhimurium [10], and Haemophilus influenza [11].

CRP is a marker for inflammation, and its levels increase during bacterial infection [12]. It is well known that while immunosuppressed patients may not develop the usual symptoms of sepsis, such as fever, elevation of CRP levels will usually be present [13,14]. The findings of a meta-analysis on a population of critically immunosuppressed patients revealed that CRP was a useful screening tool for sepsis [15].

TCZ binds soluble as well as membrane-bound IL-6 receptors, hindering IL-6 from exerting its pro-inflammatory effects, and thereby influencing CRP levels and function. IL-6 knockout mice models showed an impaired acute-phase response and were found to be vulnerable to bacterial and fungal infections [16]. 

A cumulated pool analysis of integrated safety from TCZ clinical trials on patients with RA [17] as well as real life experience point to an increased rate of serious infection up to 4.9 per 100 PY for the 8-mg/kg group [18]. It has been observed that CRP levels drop quickly after TCZ treatment has been initiated. Likewise, patients may not develop fever as a result of the suppression of the production of other proinflammatory acute-phase reactants. A number of case studies reported masking or absence of signs of infectious disease in patients treated with TCZ [19,20,21,22]. 

The objective of this study was to review nine patients treated with TCZ who were hospitalized at our center due to serious infections, and place special emphasis on the CRP status during the severe infectious event that required the index hospitalization.

## 2. Methods

We performed a retrospective chart review of patients hospitalized for serious infections who were being treated with TCZ during 2009–2018. We searched for all patients with the diagnosis of RA who were hospitalized due to serious infection and reviewed all the admissions in order to select the patients who were treated with TCZ. We also retrieved the records of all the RA and GCA patients who were treated with TCZ who were followed up in the Department of Rheumatology. The inclusion criteria were age above 18 years; ongoing intravenous (IV, 4 mg/kg and 8 mg/kg) or subcutaneous (SC, 162 mg once weekly) TCZ treatment; admission for hospitalization due to severe infection; availability of CRP levels on admission and during hospitalization; and data on duration of TCZ treatment before admission, time of hospitalization, and outcome. CRP levels of 0–5 mg/L were taken as normal. 

Ethical approval was received from the institutional review board of Tel Aviv Medical Center (Helsinki committee) that waived patient’s written informed consent for retrospective data collection.

## 3. Case Series

Nine patients with serious infections who were treated with tocilizumab were included in this case series. Each is described in order to illustrate the natural course of infectious disease during TCZ treatment and findings during hospital admission.

### 3.1. Case 1

This 59-year-old male presented with a clinical picture of seronegative RA whose main features were symmetric polyarthritis, negative rheumatoid factor (RF) and anti–citrullinated protein antibody (ACPA), and increased erythrocyte sedimentation rate (ESR) and CRP levels in 2009. He was treated with methotrexate (MTX), hydroxychloroquine, and low-dose prednisone and switched to adalimumab in 2010. In 2012, adalimumab was stopped due to increased disease activity and TCZ was initiated and resulted in prompt clinical and laboratory improvement, although he continued to suffer from low disease activity. In 2016, he developed left homonymous hemianopsia. A brain computerized tomogram (CT) revealed hypodense ischemic lesions in the basal ganglia and cerebellum. An echocardiogram and a cardiac CT revealed large vegetations on the aortic valve. At this time, the CRP levels were within normal range (0.97 mg/L). The patient was diagnosed as having endocarditis with septic embolic manifestations, and an aortic valve replacement was performed. The pathologic study of the removed aortic valve was positive for PAS-staining. The PCR confirmed the presence of Tropheryma whipplei, establishing the diagnosis of Whipple disease. He was subsequently treated with parenteral ceftriaxone for 1 month followed by maintenance therapy with oral trimethoprim-sulfamethoxazole with complete resolution of his symptoms. 

### 3.2. Case 2

This 73-year-old female had longstanding RA and was in clinical remission while being treated with TCZ for the past 9 years. Three days before admission, she developed dyspnea and cough with bloody sputum, without fever or chills. On admission, a chest CT angiogram revealed a dense consolidation in the right lower lobe compatible with infectious pneumonia. Her laboratory values on admission showed mild leucocytosis and a normal CRP level (0.03 mg/L). During hospitalization, she was treated with ceftriaxone for lobar pneumonia that led to rapid improvement in dyspnea and hemoptysis.

### 3.3. Case 3

This 55-year-old female had longstanding RA and lung nodules. She had been treated with various synthetic DMARDS and TNFα blockers, which were switched to IV TCZ 3 months before her current hospitalization. She was referred to the emergency department (ED) due to acute development of fever (~38.5 °C), productive cough with bloody sputum, and pleuritic chest pain. Her blood pressure was low (101/67 mmHg) and her oxygen saturation was 91%. Her blood tests disclosed leukocytosis up to 18.1 K, with a very slight increase of the CRP level (6.8 mg/L). Her chest X-ray showed large opacification in the left lower lobe (LLL), which was confirmed on chest CT. She was diagnosed with LLL pneumonia and started on ceftriaxone and azithromycin. On the second day of hospitalization, her CRP increased to 45.81 mg/L and decreased to 10.85 mg/L on the fifth hospitalization day in parallel with significant clinical improvement.

### 3.4. Case 4

This 67-year-old male had a 20-year history of RA that was treated over the years with csDMARDS and TNF α blockers. While on infliximab, he was diagnosed with small B cell lymphoma, which did not require treatment. The infliximab was switched to rituximab with some improvement. He subsequently started treatment with TCZ under which he developed several infectious events, such as pneumonia and herpes zoster. He was referred to our center due to the development of a red, tender, and warm occipital mass close to the cervical spine. A magnetic resonance imaging (MRI) study of the cervical spine disclosed SC collections close to the cervical spine requiring surgical draining. Cultures from the abscess and from the blood were positive for streptococcus pneumonia. His blood tests revealed mild leukocytosis (up to 13 K) and a slightly elevated CRP level (16 mg/L). 

### 3.5. Case 5

This 88-year-old female had a 35-year history of RA that was treated over the years with multiple agents, including MTX, TNFα blockers, and rituximab. She was started on TCZ 3 months before the index hospitalization, in addition to her regular use of 10 mg of prednisone/d. She had been diagnosed with an infected hematoma in her left shin 10 days before admission and was treated with oral cephalosporin with a subsequent worsening of the pain and redness in her left calf, requiring hospitalization. On admission, she had clear signs of acute cellulitis. Her blood tests disclosed leukocytosis up to 14 K, with a very slight elevation in her CRP level (7.95 mg/L). She subsequently improved after IV treatment with cefazolin.

### 3.6. Case 6

This 86-year-old female was diagnosed with giant cell arteritis 2 years earlier, based on complaints of headache and diplopia and pathological findings on temporal artery biopsy. She was treated with high-dose corticosteroids with prompt improvement. The clinical course of her disease was characterized by flares upon reducing the dosage of corticosteroids, together with clinical symptoms as well as an increased CRP level. She was started on monthly TCZ IV, at a dosage of 8 mg per kg, with prompt normalization of the CRP level. While on TCZ, she developed a purulent vaginal discharge. Hysteroscopy did not reveal an infectious process and she underwent a hysterectomy due to unabating symptoms. The pathology report revealed a cervical abscess. The symptoms resolved after the surgical procedure and treatment with antibiotics. During this period, her CRP level was normal (2.27 mg/L) after having undergone a mild increase (up to 7.5 mg/L) before the hysterectomy.

### 3.7. Case 7

This 64-year-old male had a history of recurrent middle ear infections and mastoiditis with a brain abscess at age 30 year. He was diagnosed with GCA on the basis of headache; high inflammatory markers; an abdomen CT, which revealed aortitis; positive results of right temporal artery ultrasonography; and a biopsy. Treatment with prednisone was started and azathioprine was added but his headache worsened after the dose of prednisone was reduced to 15 mg, and he was switched to SC TCZ 162 mg once weekly. Three weeks after starting treatment, he was admitted to the ED due to sudden onset of headache and fever up to 38.2 °C. His CRP level on admission was normal (0.03 mg/L). He underwent a lumbar puncture that revealed purulent cerebrospinal fluid (CSF). His clinical picture was suggestive of bacterial meningitis, and treatment with broad spectrum antibiotics was started. The CSF gram smear, culture, and BIOFIRE assay were negative. He was discharged with resolution of the infectious process after 10 days of treatment.

### 3.8. Case 8

This 62-year-old female had longstanding RA, which was in remission under TCZ treatment for more than 7 years. She was admitted to the ED due to sudden, severe abdominal pain that started 3 days earlier. There was no diarrhea or vomiting. A CT scan revealed a large amount of free air in the abdominal cavity and she underwent a laparotomy during which a perforation was observed in her colon and a large abscess was found between her uterus and recto sigma. The pathology report was consistent with diverticulitis. Her admission CRP levels were normal (0.8 mg/L). She underwent a colostomy and was discharged in a good clinical condition. The colostomy was closed 2 months later.

### 3.9. Case 9

This 76-year-old female was a heavy smoker and had a history of breast cancer that had been successfully treated 30 years before the index admission. She had been diagnosed with RA and treated with DMARDS and biologic agents for about 15 years earlier. She was switched to SC TCZ and achieved remission 2 years before this hospitalization. She was diagnosed with adenocarcinoma of the left lung and underwent left upper lobe lobectomy. Seven days after the lobectomy, she was discharged home but returned to the ED on the same day with rapid onset of dyspnea. A CT revealed bilateral lower lobe pneumonia. Her CRP level on admission was 8 mg/L. Antibiotic treatment was started, and she was intubated and mechanically ventilated. The CRP rose to 127 mg/L on the following day. A bronchoscopy was performed and it revealed a large amount of purulent discharge. She underwent a tracheostomy and her condition was observed to gradually improve. She was disconnected from mechanical ventilation 5 days later and continued to improve.

## 4. Results

We identified nine admissions of patients that were compatible with our study inclusion criteria. Seven patients were treated with TCZ for RA indication and two patients for GCA. Seven patients were treated with monthly IV TCZ and two with weekly SC TCZ. The reasons for hospitalization were pneumonia in three cases, osteomyelitis in one case, cellulitis in one case, endocarditis due to Whipple disease in one case, abscess of cervix uteri in one case, meningitis in one case, and perforated diverticulitis with intraabdominal abscess in one case. The mean CRP level on admission was 4.75 mg/L (range 0.03–16 mg/L). It was normal during the admission in four cases, mildly elevated (less than twice the upper limit) in four cases, and elevated by 3.2 (16 mg/L) from the upper laboratory limit in one case. All nine cases were diagnosed correctly on admission, and they all were observed to improve after the initiation of antibiotic treatment. Table 1 summarizes the data on the clinical and laboratory features of the nine cases described in this study.

## 5. Discussion

Patients with autoimmune diseases that are treated with TCZ may develop serious infections during the course of their treatment. Although the general suppressive effect of TCZ on CRP as an indicator of disease activity in RA is well known, its effect on the acute phase response during severe infectious episodes has been less documented. Our study is the first to summarize all the cases that have been published about infections under tocilizumab with an emphasis on inflammatory indices at presentation. Because of the specific mechanism of action of TCZ by which it blocks the Il-6 receptor, the expected rise of CRP levels during infections may be blunted and even normal despite the presence of a serious infective insult. In this study, we describe patients who were treated with TCZ and developed serious infections, such as pneumonia, meningitis, osteomyelitis, and endocarditis, and required hospitalization. The CRP levels were normal or only slightly elevated in all of these cases. Interestingly, the clinical picture was highly suggestive of the existing pathology and the diagnosis was promptly determined despite the normal CRP level in all of those patients.

Our literature search in PubMed using “tocilizumab” with special attention to cases of infectious diseases during TCZ therapy and reported CRP levels at the time of diagnosis yielded a total of 28 case reports on this specific issue (Table 2) [19,20,21,22,23,24,25,26,27,28,29,30,31,32,33,34,35,36,37,38,39,40,41,42,43]. In 15 out of 28 reported cases, CRP was normal or only slightly elevated. For illustration, Bari et al. reported a case of a 65-year-old male with RA that was well-controlled on monthly infusions of TCZ [22]. That patient developed septic arthritis in his left knee, and his CRP levels were normal despite the growth of Staphylococcus aureus in his synovial fluid and blood cultures. A Japanese study compared the clinical characteristics and severity of community-acquired pneumonia between patients with RA treated with TCZ and those treated with TNF inhibitors [44]. Those authors reported that the IL-6-inhibitor group had a significantly lower CRP level (0.09 mg/dL [range 0.02–2.5]) at diagnosis compared to the TNF-inhibitor group (6.76 mg/dL [range 0.63–15.2]). Several publications demonstrated that the risk of infections during TCZ treatment may be elevated. One British prospective observational cohort study on 19,282 patients with 46,771 person years of follow-up observed that TCZ was associated with a higher risk of serious infections compared with etanercept (HR 1.22, 95% CI 1.02 to 1.47) [45]. The possibility of higher risk of serious infections emphasizes the importance of recognizing clinical signs of infectious disease in these patients, even before their admission to hospital. In a retrospective Japanese report that used text mining, 60.7% of patients had signs/symptoms 28 days before a severe infection was diagnosed. The most frequent signs/symptoms were for infections of the skin (swelling and pain) and respiratory system (cough and pyrexia) [46]. Among 68 patients who had normal CRP levels, body temperature, and white blood cell counts, 94% had signs or symptoms of infection. 

Since CRP is not a good surrogate marker of inflammation among patients treated with TCZ, other means have been proposed. A rise of IL-6 levels during TCZ therapy may flag masked infections in patients with suppressed CRP levels. Berger et al. recently suggested that developing a kit for measuring IL-6 may help physicians to better diagnose infections in these patients [47]. In addition, in a recent study by Nagai et al., it was shown that in patients with RA treated with TCZ, using neutrophil-to-lymphocyte ratio (NLR) may be helpful for predicting bacterial infections by calculating the ratio of NLR at baseline/NLR during infection [48]. Procalcitonin, which is a marker of bacterial infections and is produced by pathways independent of IL-6, have been proposed a as preferred surrogate marker of bacterial infection during TCZ treatment [49]. Monitoring neutrophil CD64 may also be helpful for the early detection of infection in the patients treated with interleukin-6 receptor antagonists [50].

## 6. Conclusions

CRP levels may not accurately reflect the severity of infectious disease in patients who are being treated with TCZ. Although this fact may be known to rheumatologists, it should be disseminated among physicians attending patients at the ED. Prompt recognition and special attention is needed when evaluating these patients for probable infectious disease while on TCZ treatment. Interestingly, in our study, despite the impressive effect of TCZ on acute phase response, symptoms were not masked by the treatment and no significant delay in the diagnosis was made, leading in a prompt initiation of antibiotics and resolution of symptoms.

## Figures and Tables

**Table 1:** Clinical and laboratory features of the nine reported cases.

CASE	1	2	3	4	5	6	7	8	9
**Diagnosis**	RA	RA	RA	RA	RA	GCA	GCA	RA	RA
**Age**	59	73	55	67	88	86	64	62	16
**Gender**	M	F	F	M	F	F	M	F	F
**Duration of TCZ treatment**	4 years	9 years	3 months	3 years	3 months	2 months	3 weeks	7 years	2 years
**Infection diagnosed**	Whipple disease endocarditis	RLL lobar pneumonia	LLL pneumonia	Streptococcuspneumonia skin abcess	Left leg Cellulitis	Abcess of cervix uteri	Meningitis	perforated diverticulitis with intra-abdominal abscess	Bilateral pneumonia
**CRP on admission(mg/L)**	0.97	0.03	6.8	16	7.9	2.2	0.03	0.8	8
**Duration of hospitalisation, days**	>1 month	4	5	7	3	5	10	7	14
**Resolution of infection (all improved)**	After valve replacement and prolonged antibiotic Tx	Rapid	Rapid	Gradual	Gradual	after hysterectomy	Gradual	After colostomy and antibiotic Tx	Gradual

RA, rheumatoid arthritis; GCA, giant cell arteritis; TCZ, tocilizumab; CRP, C-reactive protein; RLL, right lower lobe; LLL, left lower lobe; Tx, treatment.

**Table 2:** Clinical characteristics of reported cases of infectious diseases, during TCZ therapy in which CRP levels were noted.

Authors and Year	TCZ Tx Duration	Age	Sex	TCZ DOSE	Type of Infection	CRP at Diagnosis	Indication for TCZ
Yoshiyuki Arinuma et al., 2011	2 years	64	M	8 mg/kg q 4 weeks, IV	Legionella Pneumonia	14.54 mg/dL	RA
Hidehiro Honda et al., 2009	8 weeks	69	F	8 mg/kg q 2 weeks, IV	allergic bronchopulmonary aspergillosis	0.0 mg/dL	RA
M. Yéléhé-Okoumaa et al., 2016	13 months	80	F	8 mg/kg q 4 weeks, IV	Erysipelas	1 mg/dL	RA
Diana Rosa-Gonc alves et al., 2016	3 years	66	F	8 mg/kg q 4 weeks, IV	Necrotizing fasciitis	7.46 mg/dL	RA
Daisuke Kobayashi et al., 2013	3 years	53	F	8 mg/kg q 4 weeks, IV	Mycobacterium abscessus pulmonary Infection	0.01 mg/dL	RA
Manabe et al., 2017	3 years	76	F	400 mg q 4 weeks, IV	Necrotizing soft tissue infection	10.73 mg/dL	RA
Komura et al., 2015	19 days	54	F	N/A	CMV reactivation hepatitis	5.4 mg/dL	RA
Yoshida et al., 2011	12 months	65	F	8 mg/kg q 4 weeks, IV	Necrotizing fasciitis	0.04 mg/dL	RA
Yamate et al., 2019	2 months	67	F	162 mg q 2 weeks, SC	Disseminated nocardiosis	3.52 mg/dL	RA
Williams et al., 2019	6 months	81	F	162 mg q 1 week, SC	Pseudomonas meningoencephalitis	1 mg/dL	GCA
Kenji Okumura et al., 2011	N/A	72	F	8 mg/kg q 4 weeks, IV	Aeromonas hydrophila sepsis	2.16 mg/dL	RA
Tsujimoto et al., 2015	2 years	69	F	8 mg/kg q 4 weeks, IV	Pyogenic spondylodiscitis	13.3 mg/dL	RA
de Kruif, 2012	6 months	63	F	8 mg/kg q 4 weeks, IV	Streptococcal lung abscesses	Normal at presentation	Takayasu arteritis
Raine et al., 2013	N/A	61	M	N/A	Pyomyositis	<0.5 mg/dL	RA
J. Razanamahery et al., 2020	6 months	50	M	0.7 mg/kg/month, IV	Whipple disease	2 mg/dL	RA
Reisinger et al., 2020	2 weeks	46	F	N/A	Tuberculosis sepsis	4.03 mg/dL	systemic sclerosis and SLE overlap
van de Sande et al., 2011	2 months	59	F	800 mg q 4 w, IV	Necrotizing fasciitis	Normal	RA
Wakabayashi et al., 2013	9 months	71	M	N/A	Polyarticular septic arthritis	7.15 mg/dL	RA
Bari et al., 2013	N/A	65	M	8 mg/kg q 4 weeks, IV	*S. aureus* septic arthritis	Normal	RA
Yanagawa et al., 2012	N/A	78	F	80 mg q 4 weeks, IV	Pneumonia	<0.3 mg/dL	RA
Conway et al., 2017	N/A	70	M	162 mg q 1 week, SC	Septic arthritis	<0.1 mg/dL	RA
Hirao et al., 2011	7 months	62	F	8 mg/kg q 4 weeks, IV	Cellulitis	<0.04 mg/dL	RA
Hirao et al., 2011	5 months	49	F	8 mg/kg q 4 weeks, IV	Cellulitis	<0.04 mg/dL	RA
Fujiwara et al., 2008	11 months	68	M	8 mg/kg q 4 weeks, IV	Pneumonia	0.55 mg/dL	RA
Fujiwara et al., 2008	8 months	68	M	8 mg/kg q 4 w	Pneumonia	2.3 mg/dL	RA
Nguyen et al., 2013	N/A	68	F	N/A	disseminated *S. aureus* infection	1.4 mg/dL	RA
Nguyen et al., 2013	N/A	63	M	N/A	disseminated *S. aureus* infection	4.5 mg/dL	RA
Nguyen et al., 2013	N/A	59	M	N/A	disseminated *S. aureus* infection	3.6 mg/dL	RA

## Data Availability

The data presented in this study are openly available.
